# Mapping the patterns of cortical thickness in single- and multiple-domain amnestic mild cognitive impairment patients: a pilot study

**DOI:** 10.18632/aging.102362

**Published:** 2019-11-22

**Authors:** Pan Sun, Wutao Lou, Jianghong Liu, Lin Shi, Kuncheng Li, Defeng Wang, Vincent CT Mok, Peipeng Liang

**Affiliations:** 1 Department of Medicine and Therapeutics, The Chinese University of Hong Kong, Hong Kong, China; 2 Department of Neurology, Xuanwu Hospital, Capital Medical University, Beijing, China; 3 Department of Imaging and Interventional Radiology, The Chinese University of Hong Kong, Hong Kong, China; 4 BrainNow Research Institute, Shenzhen, China; 5 Department of Radiology, Xuanwu Hospital, Capital Medical University, Beijing, China; 6 Beijing Key Lab of MRI and Brain Informatics, Beijing, China; 7 School of Instrumentation Science and Opto-electronics Engineering, Beihang University, Beijing, China; 8 Therese Pei Fong Chow Research Centre for Prevention of Dementia, The Chinese University of Hong Kong, Hong Kong, China; 9 Beijing Key Laboratory of Learning and Cognition, School of Psychology, Capital Normal University, Beijing, China

**Keywords:** cortical thickness, Alzheimer’ disease, amnestic mild cognitive impairment, multiple-domain, single-domain

## Abstract

Amnestic mild cognitive impairment (aMCI) is considered as a transitional stage between the expected cognitive decline of normal aging and Alzheimer’s disease (AD). Structural brain difference has shown the potential in cognitive related diagnosis, however cortical thickness patterns transferred from aMCI to AD, especially in the subtypes of aMCI, is still unclear. In this study, we investigated the cortical thickness discrepancies among AD, aMCI and normal control (NC) entities, especially for two subtypes of aMCI - multiple-domain aMCI (aMCI-m) and single-domain aMCI (aMCI-s). Both region of interest (ROI)-based and vertex-based statistical strategies were performed for group-level cortical thickness comparison. Spearman correlation was utilized to identify the correlation between cortical thickness and clinical neuropsychological scores. The result demonstrated that there was a significant cortical thickness decreasing tendency in fusiform gyrus from NC to aMCI-s to aMCI-m to finally AD in both left and right hemispheres. Meanwhile, the two subtypes of aMCI showed cortical thickness difference in middle temporal gyrus in left hemisphere. Spearman correlation indicated that neuropsychological scores had significant correlations with entorhinal, inferior temporal and middle temporal gyrus. The findings suggested that cortical thickness might serve as a potential imaging biomarker for the differential diagnosis of cognitive impairment.

## INTRODUCTION

As one of the most common neurodegenerative disorder, there is still not any effective treatment to stop the brain changes caused by Alzheimer’s disease (AD) nowadays [1]. Therefore, investigating early-detection and intervention approaches for early-stage AD symptoms are vitally important. Amnestic mild cognitive impairment (aMCI), characterized by memory impairment between normal aging and early dementia [2], is thought to reveal a high risk of progression to dementia, especially to AD [3]. Much attention has been centered on studying aMCI [4] and its two subtypes – single-domain of aMCI (aMCI-s), which is characterized by memory impairment, whereas general cognitive function and daily activity remain less affected [5], and multiple-domain of aMCI (aMCI-m), which exhibits memory loss as well as at least one other cognitive domain decline [6]. It has been considered that aMCI-m and aMCI-s are theoretically different entities, however, only a few studies investigated the structural brain difference between the two aMCI subtypes [7, 8].

Neuroimaging methods provide a non-invasive way of delineating brain structure and function *in vivo* and have been utilized for the study on the differential between aMCI-m and aMCI-s. Resting state abnormalities were observed between the two subtypes of aMCI using functional MRI [9]. Also, fractional anisotropy (FA) and mean diffusivity (MD) alteration were compared using diffusion tensor imaging (DTI) [10]. Connectivity damage difference was discussed between aMCI-m and aMCI-s using positron emission tomography (PET) [11]. As for the structural neuroimaging analysis for the two subtypes of aMCI, atrophy and volume of the cortical gray matter or manually defined regions of interest (ROIs) have been discussed [12, 13]. These studies are of great importance to portray the progression pathway occurring from normal aging of brain to aMCI. Cortical thickness enabled precise measurement [14, 15] and it was widely reported to have differentials between AD and NC groups in many cortical regions [16–19]. Besides, the atrophy pattern of cortical thickness was proven to reflect increasing disease severity [20] and demonstrated regional heterogeneity in aMCI patients [8]. However, the cortical thickness difference among AD, aMCI and NC entities, especially for the two subtypes of aMCI - aMCI-m and aMCI-s, is still not clear. More work should be conducted for probing into the differential diagnosis of aMCI-m and aMCI-s based on objective structural cortical thickness characteristics.

The aim of our study was to identify the structural cortical thickness difference among AD, aMCI-m, aMCI-s and NC groups by both ROI-based and vertex-based methods. Based on previous studies on AD and aMCI subjects [21, 17], we hypothesized that both aMCI-m and aMCI-s groups might have significant cortical thickness decrease comparing to NC group, while AD group would be more likely to exhibit cortical thickness decrease than both aMCI-m and aMCI-s groups. In addition, the two subtypes of aMCI might demonstrate differential thickness decrease patterns in certain cortical regions (*e.g.* anterior cingulate region), which have been approved to have cortical thickness reduction in AD patients comparing to NC subjects [22].

## RESULTS

### Demographic and neuropsychological information

The demographic and neuropsychological details of the participants used in this study are shown in Table 1. There was no significant difference in age, gender and education for AD, aMCI-m, aMCI-s and NC groups whereas all the neuropsychological assessments showed significant difference among the four groups. The AVLT, MMSE and MoCA scores were quite approximated between aMCI-m and aMCI-s whereas the TMT and BNT scores indicated group-level difference between the two groups.

**Table 1:** Details of the participant study groups; mean ± standard deviation is given. AVLT, MMSE and MoCA are based on number correct and TMT is based on seconds.

**Characteristic**	**AD (n = 15)**	**aMCI-m (n = 18)**	**aMCI-s (n = 25)**	**NC (n = 15)**	**F value**	**p value**
**Age (year)**	70.40±9.19	72.17±6.46	71.88±6.15	68.80±6.47	0.827	0.484
**Gender (M/F)**	6/9	10/8	12/13	8/7	0.292	0.831
**Education**	7.87±4.02	10.17±3.90	8.52±3.73	10.53±3.70	1.86	0.144
**AVLT ^a^**	12.53±8.19	29.39±10.93	28.00±5.52	47.93±9.71	43.367	<0.001*
**MMSE ^b^**	16.87±7.64	24.50±3.96	24.64±2.72	28.53±1.60	19.205	<0.001*
**MoCA ^c^**	12.20±5.91	20.39±4.38	19.96±3.93	26.13±2.07	27.235	<0.001*
**TMT ^d^**	257.40±46.28	114.39±29.49	80.60±22.97	84.40±34.43	105.299	<0.001*
**BNT ^e^**	13.40±6.80	23.33±2.17	27.64±1.22	29.00±0.93	71.172	<0.001*

*indicates group-level significant different level attributes obtained by one-way ANOVA (p < 0.05, uncorrected).

### Averaged cortical thickness

**Figure 1:**
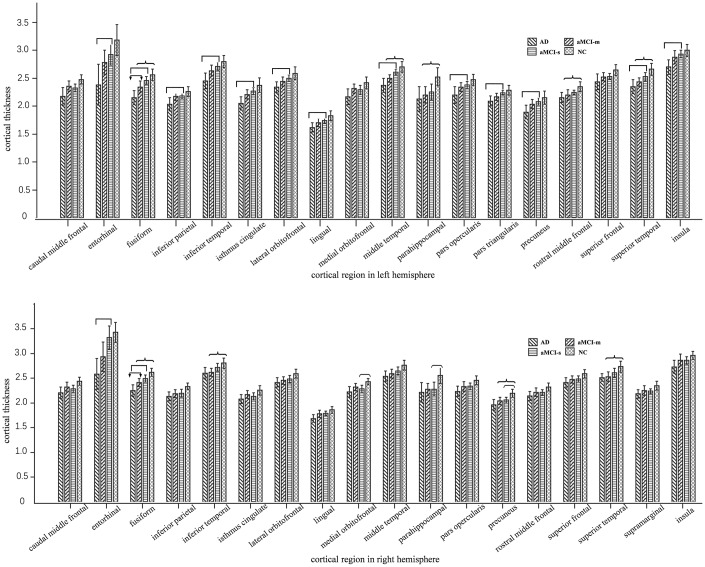
**Plots of cortical thickness values with significant group differences in one-way ANOVA.** The above one is left hemisphere and the below one is right hemisphere. We marked out the significantly different cortical thickness regions with Tukey’s multiple comparison test (p < 0.05) between AD and aMCI-m group (with 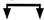 marker), AD and aMCI-s group (with 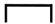 marker), aMCI-m and NC group (with 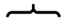 marker), aMCI-s and NC group (with 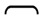 marker).

In the ANOVA comparison among the four groups, fusiform was observed significant cortical thickness decreasing tendency from NC to aMCI-s to aMCI-m to AD both in left and right hemispheres as shown in Figure 1 (Supplementary Table 1). For left hemisphere, F = 12.479, p < 0.001, with Tukey’s multiple comparison test: p value of AD versus aMCI-m was 0.035, p value of AD versus aMCI-s was less than 0.001, p value of AD versus NC was less than 0.001, p value of aMCI-m versus NC was 0.012. For right hemisphere, F = 12.147, p < 0.001, with Tukey’s multiple comparison test: p value of AD versus aMCI-m was 0.049, p value of AD versus aMCI-s was less than 0.001, p value of AD versus NC was less than 0.001, p value of aMCI-m versus NC was 0.006. For the aMCI-m group, there was significant cortical thickness decrease in certain cortical areas compared with NC group after Tukey’s multiple comparison, such as left hemisphere middle temporal (p = 0.003), parahippocampal (p = 0.038), rostral middle frontal (p = 0.025), superior temporal (p = 0.004) and right hemisphere inferior temporal (p = 0.029), precuneus (p = 0.036) and superior temporal (p = 0.019). As for the aMCI-s group, right hemisphere medial orbitofrontal (p = 0.045) and precuneus (p = 0.044) indicated significant cortical thickness reduce compared with NC group. There was no ROI-based significant cluster between aMCI-m and aMCI-s groups.

### ROI-based cortical thickness correlation with neuropsychological measurements

**Figure 2:**
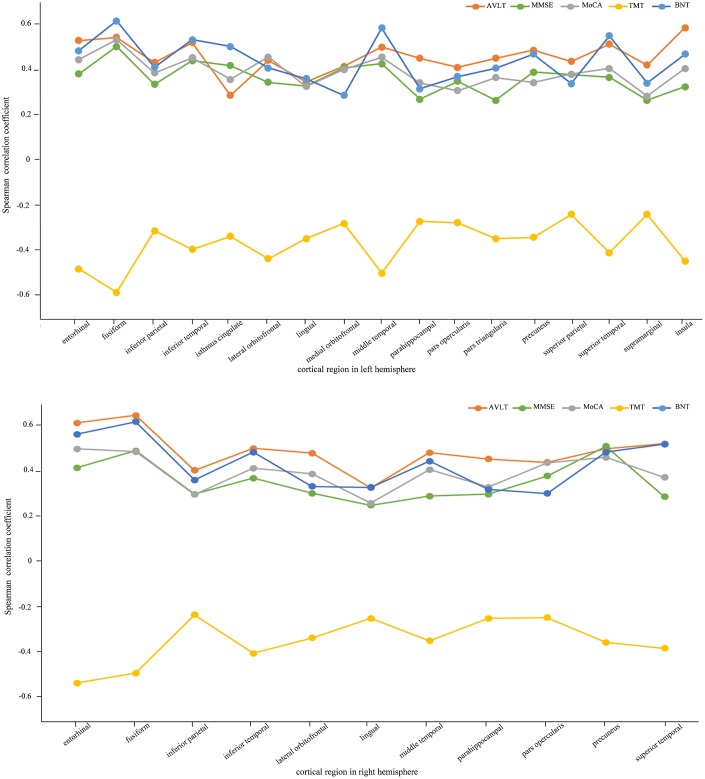
**Spearman correlation between cortical thickness and neuropsychological measurements for AD, aMCI-m, aMCI-s and NC groups.** The figures list the high correlation cortical regions with corresponding neuropsychological measurements (p < 0.05). The above one is left hemisphere and the below one is right hemisphere.

In order to investigate the relationship between cortical thickness and neuropsychological measurements, Spearman correlation was utilized. The averaged cortical thicknesses of the 62 cortical areas defined by DKTatlas40 were utilized as the independent variables and the five neuropsychological measurements, *i.e*. AVLT, MMSE, MoCA, TMT and BNT, were used as dependent variables respectively. Figure 2 (Supplementary Table 2) shows the cortical regions significantly correlated with all the neuropsychological assessments (p < 0.05). The cortical thickness had positive correlations with AVLT, BNT, MMSE and MoCA whereas negative relationship with TMT, which was in accordance with previous study [23]. The absolute values of correlation coefficients were range from 0.240 to 0.622. As for the aMCI-m and aMCI-s groups, neuropsychological AVLT, TMT and BNT indicated significant correlations with multiple cortical areas, which included entorhinal, inferior temporal and middle temporal gyrus shown in Table 2.

**Table 2:** Spearman correlation between cortical thickness and neuropsychological measurements for aMCI-m and aMCI-s groups.

	**left hemisphere**			**right hemisphere**		
	**cortical region**	**r**	**p**	**cortical region**	**r**	**p**
**AVLT**	inferior temporal	0.33	0.03	entorhinal	0.46	< 0.01
	parahippocampal	0.38	0.01	fusiform	0.33	0.03
	pars orbitalis	0.38	0.01	inferior temporal	0.44	< 0.01
	pars triangularis	0.38	0.01	lateral orbitofrontal	0.40	< 0.01
	precentral	0.30	0.05	middle temporal	0.34	0.03
	rostral middle frontal	0.34	0.03	rostral middle frontal	0.35	0.02
	superior parietal	0.38	0.01	insula	0.47	< 0.01
	insula	0.51	< 0.01			
**MMSE**				precuneus	0.34	0.03
**MoCA**	lateral orbitofrontal	0.30	0.05		0.31	0.01
**TMT**	entorhinal	-0.35	0.02	entorhinal	-0.36	0.02
	middle temporal	-0.30	0.05	inferior temporal	-0.32	0.04
	pars orbitalis	-0.41	< 0.01		-0.26	0.03
	posterior cingulate	-0.35	0.02		-0.29	0.02
**BNT**	fusiform	0.35	0.02	entorhinal	0.43	< 0.01
	inferior temporal	0.35	0.02	fusiform	0.34	0.03
	middle temporal	0.38	0.01	inferior temporal	0.38	0.01
	superior temporal	0.34	0.03	superior temporal	0.35	0.02

The table lists the high correlation cortical regions with corresponding neuropsychological measurements (p < 0.05).

### Vertex-based group cortical thickness analysis

All the AD, aMCI-m and aMCI-s groups showed significantly decreased cortical thickness in left and right hemispheres compared with NC group, whereas their decrease patterns were distinctive. For the fusiform gyrus in left hemisphere, cortical thickness of AD group decreased than aMCI-m, aMCI-s and NC groups, meanwhile aMCI-m group cortical thickness reduced than NC group. For the fusiform gyrus in right hemisphere, cortical thickness of AD group decreased than aMCI-s and NC groups. For the middle temporal gyrus in left hemisphere, cortical thickness of AD group decreased than aMCI-s and NC groups, meanwhile aMCI-m group cortical thickness reduced than aMCI-s and NC. For the entorhinal gyrus, aMCI-m cortical thickness decreased than NC group. The superior frontal and superior parietal decreased in aMCI-s group than NC group. No thickening cortical region was detected for the patient group than NC group. The statistically significant clusters are shown in Figure 4 and the details of the clusters are listed in Table 3.

**Table 3:** Significant statistical analysis clusters for AD, aMCI-m, aMCI-s and NC (Monte Carlo simulation corrected for multiple comparisons).

**hemisphere**		**cluster**	**cluster size(mm^2^)**	**Talairach**			**CWP**	**cortical region**
				**x**	**y**	**z**		
**AD vs aMCI-m**	left	1	1,152.2	-40.5	-52.8	-14.1	< 0.01	fusiform
**AD vs aMCI-s**	left	1	6,997.7	-35.3	-50.8	-12.4	< 0.01	fusiform
		2	2,350.9	-7.0	-49.8	18.5	< 0.01	isthmus cingulate
		3	2,321.4	-42.2	-15.7	-9.3	< 0.01	superior temporal
		4	1,146.2	-33.5	12.4	23.0	0.01	caudal middle frontal
	right	1	2,398.8	19.3	-83.7	-2.2	< 0.01	lateral occipital
**AD vs NC**	left	1	17,196.7	-31.0	-52.2	-3.7	< 0.01	fusiform
		2	6,979.2	-24.7	30.5	29.1	< 0.01	rostral middle frontal
		3	2,626.7	-13.5	-40.9	31.5	< 0.01	isthmus cingulate
		4	2,239.5	-21.5	27.7	-11.7	< 0.01	lateral orbitofrontal
	right	1	9,284.5	35.2	4.7	-13.4	< 0.01	insula
		2	6,581.7	33.7	14.9	22.3	< 0.01	caudal middle frontal
		3	2,852.0	20.3	32.7	31.2	< 0.01	superior frontal
		4	2,363.5	43.1	-48.7	18.7	< 0.01	inferior parietal
**aMCI-m vs aMCI-s**	left	1	1,176.7	-61.9	-35.5	-9.4	< 0.01	middle temporal
**aMCI-m vs NC**	left	1	2,482.3	-43.4	-47.4	12.8	< 0.01	bankssts
		2	1,761.0	-36.9	-0.8	-12.8	< 0.01	insula
		3	1,319.3	-32.0	-38.1	-7.6	< 0.01	parahippocampal
		4	928.8	-39.1	27.8	15.1	0.04	rostral middle frontal
	right	1	1,357.4	50.0	4.4	18.9	< 0.01	precentral
		2	1,312.5	10.6	-54.1	18.7	< 0.01	precuneus
		3	913.2	28.3	-60.5	-8.2	0.03	fusiform
**aMCI-s vs NC**	left	1	1,646.2	-19.3	29.1	33.5	< 0.01	superior frontal
	right	1	1,400.1	24.1	-52.2	48.2	< 0.01	superior parietal

The size of the significant cluster, Talairach coordinates and cortical areas corresponding to the most significant vertex within the cluster, cluster-wise p value (CWP) are shown.

**Figure 3:**
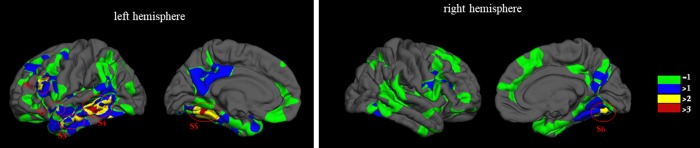
**Significant frequency for the vertex-based statistical analysis.** Green indicated the areas showed significant clusters in one comparison for the four group comparisons and blue indicated the areas showed statistically significant in at least two group comparisons.

Based on the vertex-based significant analysis results, we mapped the statistically significant cortical clusters at p-value < 0.05 and summarized the significant frequency at vertex in fsaverage surface as shown in Figure 3 and Table 4. Then the averaged cortical thicknesses in areas which showed statistical significance in at least three between-group comparisons (the cortical areas marked with yellow and red color) were computed to probe the relationship between cortical thickness and clinical scores in aMCI-m and aMCI-s groups. Here, TMT and BNT scores were used to calculate the Spearman correlation. The statistical analysis results indicated that the cortical thickness in left hemisphere middle temporal and left hemisphere superior temporal correlated with both TMT and BNT scores for aMCI-m and aMCI-s groups.

**Table 4:** Spearman correlation between clinical scores and the cortical thickness in statistically significant frequency group comparison over two times.

**score**	**s1**		**s2**		**s3**		**s4**		**s5**		**s6**	
	**r**	**p**	**r**	**p**	**r**	**p**	**r**	**p**	**R**	**p**	**r**	**P**
**TMT**	0.22	0.16	0.24	0.13	0.48	< 0.01*	0.43	0.01*	0.25	0.11	0.21	0.17
**BNT**	-0.18	0.26	-0.36	0.02*	-0.29	0.06	-0.37	0.02*	-0.25	0.11	0.03	0.84

*indicates significant correlation at 0.05 level; r is Spearman correlation coefficient.

## DISCUSSION

In the current study, cortical thickness difference was explored among AD, aMCI-m, aMCI-s and NC subjects. The ROI-based analysis showed that the cortical thicknesses of some areas (*e.g.* left hemisphere fusiform and middle temporal, right hemisphere fusiform) showed group-level significant difference among the four groups. The Spearman correlation test for aMCI-m and aMCI-s groups indicated that the cortical thickness was highly correlated with neuropsychological scores in entorhinal, fusiform and middle temporal gyrus. In addition, the vertex-based group analysis showed a decrease tendency from NC to MCI to final AD.

In the ROI-based analysis, four cortical areas, respectively left hemisphere fusiform (F = 12.48, p < 0.01), middle temporal (F = 12.48, p < 0.01) and right hemisphere fusiform (F = 12.15, p < 0.01), entorhinal (F = 8.34, p < 0.01) expressed high group-level difference in ANOVA (as shown in Figure 5). Previous studies have shown that temporal gyrus atrophy is a sensitive marker of aMCI [24, 25]. In our study, the mean cortical thickness values of left hemisphere fusiform, middle temporal and right hemisphere fusiform, entorhinal showed a decrease trend from NC to aMCI-s to aMCI-m to AD. These findings suggest aMCI-m is more likely progress to AD and it maybe a transitional stage between aMCI-s and AD [26]. However, no cortical area was identified as significantly different between aMCI-m and aMCI-s in this ROI-based cortical thickness comparison. The potential causal effects might partly be that for a certain cortical ROI area, a part of it was suffered from the cortical thickness change for aMCI-m or aMCI-s patients. In this case, the statistical analysis utilizing the whole ROI might reduce the local significance between aMCI subtypes. Besides, there might be inhomogeneity in cortical thickness within aMCI-m or aMCI-s group. This might also result in not significant statistical outcomes even if the averaged within-group cortical thickness was largely different.

**Figure 4:**
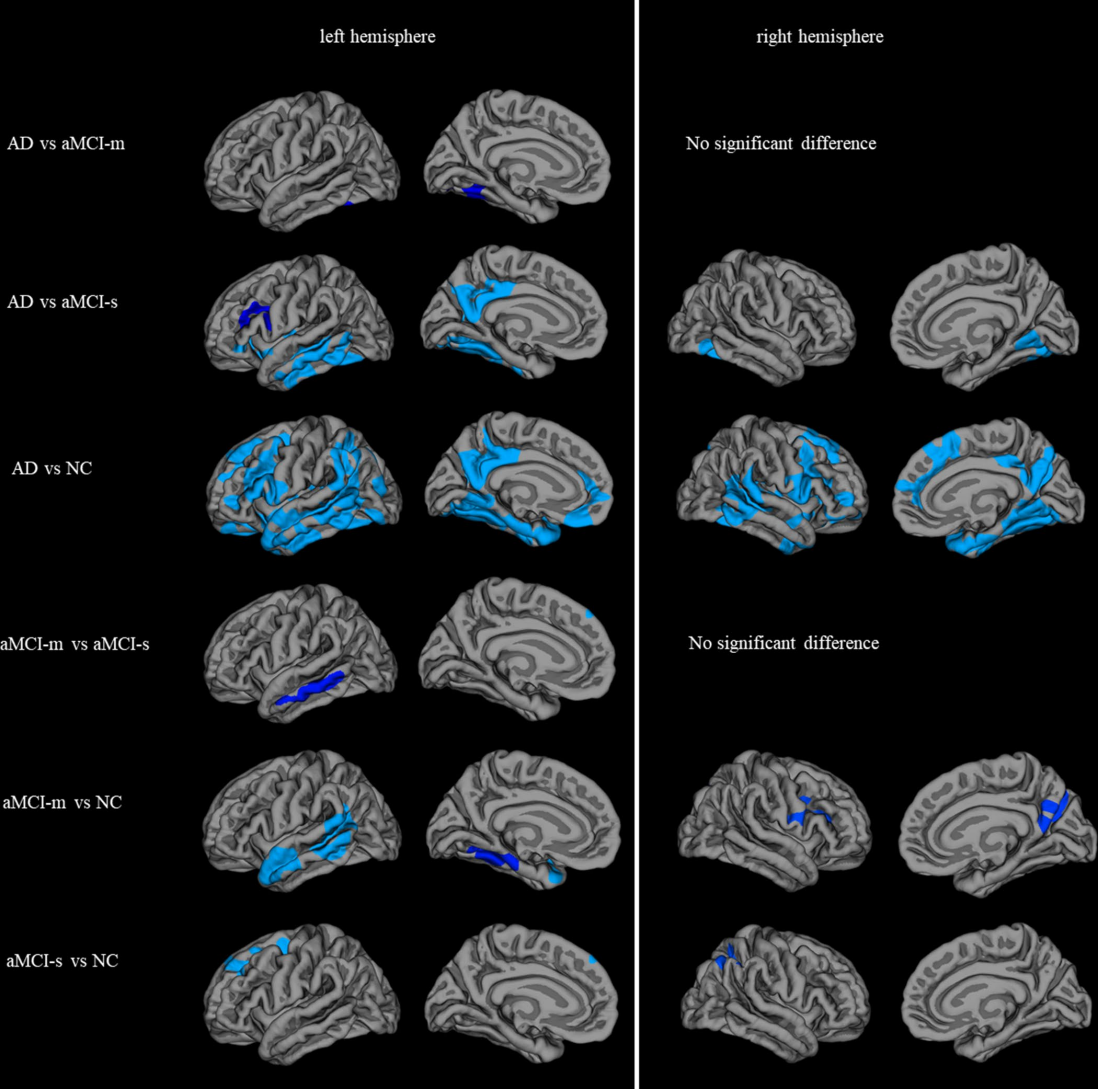
**Statistically significant brain regions obtained by group comparisons between the AD, aMCI-m, aMCI-s and NC groups (Monte Carlo simulation corrected for multiple comparisons).** The significance was -log(p) instead of straight p value for display purpose.

In the Spearman correlation for ROI-based cortical thickness with neuropsychological scores among the four groups, multiple cortical areas presented significant correlations with neuropsychological scores in both left and right hemispheres. These areas included temporal lobe (*e.g.* entorhinal, fusiform, parahippocampal, inferior temporal, middle temporal, superior temporal), parietal lobe (*e.g.* inferior parietal, precuneus), occipital lobe (*e.g.* lingual, lateral occipital) and frontal lobe (*e.g.* pars opercularis). The further analysis between aMCI-m and aMCI-s also indicated that temporal lobe (*e.g.* fusiform, entorhinal, inferior temporal, superior temporal) was significantly correlated with neuropsychological scores. These results suggested a decrease of cortical thickness in the specific cortical areas were correlated with poorer performance on neuropsychological tests [27]. However, the neuropsychological scores were challenging to differentiate different aMCI patients in clinical. One of the possibilities is that the scores are integrated by multiple factors and they are not sensitive enough to reflect the changes. Probing into the cortical thickness in specific areas especially the temporal lobe might contribute to diagnoses of diversity in different aMCI patients.

**Figure 5:**
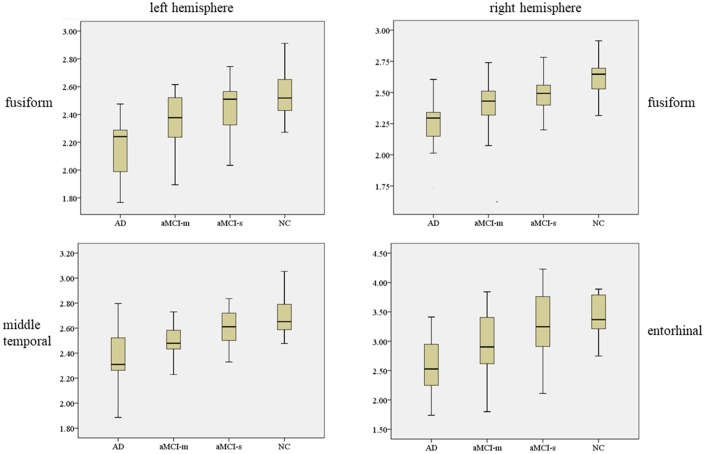
**The mean ± standard deviation values for the cortical thickness in left hemisphere fusiform, middle temporal and right hemisphere fusiform, entorhinal.**

The Spearman correlation analysis between TMT, BNT scores and cortical areas, which were vertex-based statistical significance in at least three between-group comparisons, middle and superior temporal showed correlations with the two clinical scores for aMCI-m and aMCI-s groups. The results coincide with previous longitudinal study [28] stating that temporal gyrus occurred great changes to clinically probable AD criteria over time, whereas superior temporal cortex, together with the anterior frontal and inferior parietal cortices, showed changes in later aMCI stage. Besides, there were more statistically significant group comparison clusters in left hemisphere than right hemisphere. This suggested left hemisphere might be more subjected to damage for aMCI and AD. The different roles of the two hemispheres may contribute to explain this asymmetric distribution in these cortical sub-regions [29]. Recent functional MRI studies also detected a decreased lateralization of sensorimotor and attention [30, 31].

Structures within temporal lobe have been frequently proved correlating with AD decline for their critical role in the formation of long-term memory [32]. And the temporal lobe was reported in a number of critical abilities including processing of intention, faces, emotion, speech [33]. Atrophy of the structures within temporal lobe may be correlated with the abnormality of related function. In previous study [32], entorhinal cortex atrophy was proven closely relating to cognitive function and thinner entorhinal cortex was reported in AD patients compared with NC. The entorhinal cortex and hippocampus, as well as the anatomically related perirhinal and parahippocampal cortices, are components of the medial temporal lobe (MTL) memory system [34, 35], which is essential for declarative memory [36]. Fusiform cortex was thought to be involved in higher order visual processing and middle temporal gyrus was correlated with emotional contagion [33]. Cortical thickness was considered affecting the MTL very early, soon after extending to the remainder of the cortex along a temporal-parietal-frontal trajectory, while motor areas are generally spared until late disease stages [34, 37]. In line with the multiple function decline in cognitive patients, we observed the cortical thickness reduction in multiple temporal lobe subregions.

There were still some limitations in this study. First, cortical thickness is not the only factor correlating with cognition, as other factors, *e.g.* vascular condition and increased white matter hyperintensity (WMH) volume, might also affect the cognition [38]. Identifying the cortical decrease difference could help to understand the different developmental stage whereas our present research findings still could not be treated as a quantitative biomarker for diagnosis. Second, the sample size used in this study was not large and the samples were unbalanced that there were more aMCI-s subjects than aMCI-m subjects, whereas previous studies reported that aMCI-s was less common than aMCI-m [8]. Larger participant samples, especially for the two subtypes of aMCI, would be enrolled in the further. Besides, in the current study, cortical thickness difference was examined cross-sectionally and future studies should contain longitudinal comparison for aMCI-s and aMCI-m respectively.

Taken together, we quantitatively explored the cortical thickness difference among AD, aMCI-m, aMCI-s and NC subjects, as well as the relationship between cortical thickness and clinical cognitive impairment scores. The findings suggested that cortical thickness might be served as a potential imaging biomarker for the differential diagnosis of cognitive impairment. Also, different cortical thickness decreasing pattern might provide diagnostic symptom in early-stage AD. We expect that our pilot study in comparison of cortical thickness between aMCI-m and aMCI-s could be beneficial to the biomarker exploration of cognitive impairment diagnosis.

## MATERIALS AND METHODS

### Participants

We initially enrolled 93 participants including 19 AD, 22 aMCI-m, 29 aMCI-s and 23 NC subjects from the Department of Neurology of Xuanwu Hospital, Capital Medical University. Ethical permission was approved by local committee, and written informed consents were obtained from all participants or their relatives before the MRI scan. The battery of neuropsychological assessments was performed by two experienced neurological doctors according to the international standard criteria [39, 40], which involved Auditory-Verbal Learning Test (AVLT) score, Mini-Mental State Examination (MMSE) score, Montreal Cognitive Assessment (MoCA) score, Trail-Making Test (TMT) score, and Boston Naming Test (BNT) score. AVLT, MMSE, MoCA and BNT scores are based on the number correct, whereas TMT is based on the reaction time. Higher number correct indicates better cognitive performance [6, 41], whereas longer reaction time indicates worse cognitive performance [42]. 15 participates were excluded for age factors (we concentrated on the participants between 60 and 85 years old in this study), 2 participants were excluded for lack of neuropsychological scores, and 3 subjects were eliminated for abnormal segmentation results. Finally, 73 subjects were utilized for the cortical thickness analysis and their demographic and neuropsychological details were shown in Table 1.

### MRI acquisition

MRI data acquisition was performed on a 3-Tesla scanner (Siemens Medical Solutions, Erlangen, Germany). Foam padding and headphones were utilized to restrict head motion and reduce scanner noise. The 3D T1-weighted anatomical image was acquired with a magnetization-prepared rapid gradient echo (MPRAGE) method with the following parameters: repetition time (TR) / echo time (TE) / inversion time (TI) / flip angle (FA) = 1900 ms / 2.2 ms / 900 ms / 9°, acquisition matrix = 224 × 256 × 176, voxel size = 1 × 1 × 1 mm^3^.

### Structural image processing

The structural MPRAGE imaging data was processed using Freesurfer [43] and the standard processing procedures were performed, which including intensity normalization, removal of non-brain tissue [44], automated Talairach transformation, tissue segmentation of grey matter (GM) volumetric structures and subcortical white matter (WM) [45], tessellation of the GM-WM boundary, automated topology correction [46], and surface deformation to optimally place the GM-WM and GM-cerebrospinal fluid borders at the location where the greatest shift in intensity defines the transition to the other tissue class [47]. Then, a spherical atlas (fsaverage) [48] provided by Freesurfer was applied as the common space to match cortical geometry across subjects. Steps included surface inflation [49], registration to fsaverage space, parcellation of the cerebral cortex into units based on gyral and sulcal structure [50] and creation of surface based data. Cortical models and the results of segmentation were quality checked manually using Freeview [51]. Finally, spatial smoothing using a 10 mm full-width at half-maximum Gaussian kernel was carried out in fsaverage space for each subject for reducing noise. Consequently, the vertex-based statistical analysis could be performed vertex by vertex across whole-brain at group-level since the vertexes on the fsaverage space were spatially co-registered for all subjects.

### Cortical thickness statistical analysis

For assumption-free ROI-based statistical analysis, we parcellated the cortical region by the DKTatlas40 [52] atlas in fsaverage space, which defined the gyrus as frontal, parietal, temporal occipital and cingulate including 62 sub-regions (31 areas for left hemisphere and right hemisphere respectively). One-way ANOVA with Tukey’s multiple comparison test was applied to demonstrate the significant group-level different ROIs using IBM SPSS version 22. Simultaneously, Spearman correlation was utilized to probe the relationship between regional cortical thickness and AVLT, MMSE, MoCA, TMT, BNT scores respectively. As for the vertex-based statistical comparison, we analyzed the smoothed cortical thickness data in fsaverage space to compare the group-level difference. In this paper, we used query design estimate contrast (QDEC) [53] to complete the vertex-based group-level cortical statistical analysis for left and right hemispheres respectively. Generalized linear model (GLM) [54] was utilized for the significance testing vertex-by-vertex for each hemisphere. A significance threshold of p-value < 0.05 was adopted (Monte Carlo simulation corrected for multiple comparisons). The statistical results were overlaid onto semi-inflated cortical surfaces for revealing group-level different areas.

## Supplementary Material

Supplementary Tables
