# Supplementary material for: Mapping the patterns of cortical thickness in single- and multiple-domain amnestic mild cognitive impairment patients: a pilot study
Source: Aging (Albany NY). 2019 Nov 22;11(22):10000–15. doi: 10.18632/aging.102362 (PMC6914405; doi:10.18632/aging.102362)
Supplement: Supplementary Tables [file aging-11-102362-s001.pdf]

SUPPLEMENTARY TABLES

Supplementary Table 1. One-way ANOVA with Tukey's multiple comparison results. The above one is left hemisphere and the below one is right hemisphere.

	one-way ANOVA		Tukey's multiple comparison					
	F value	p value	AD vs aMCI-m	AD vs aMCI-s	AD vs NC	aMCI-m vs aMCI-s	aMCI-m vs NC	aMCI-s vs NC
**caudal middle frontal**	5.95	< 0.01	0.05	0.09	< 0.01*	0.97	0.31	0.10
**entorhinal**	6.56	< 0.01	0.12	0.01*	< 0.01*	0.81	0.12	0.41
**fusiform**	12.48	< 0.01	0.04*	< 0.01*	< 0.01*	0.23	0.01*	0.40
**inferior parietal**	5.09	< 0.01	0.08	0.04*	< 0.01*	> 0.99	0.42	0.44
**inferior temporal**	8.01	< 0.01	0.06	< 0.01*	< 0.01*	0.62	0.12	0.59
**isthmus cingulate**	8.44	< 0.01	0.07	< 0.01*	< 0.01*	0.71	0.06	0.30
**lateral orbitofrontal**	5.80	< 0.01	0.32	0.02*	< 0.01*	0.67	0.08	0.42
**lingual**	5.76	< 0.01	0.29	0.04*	< 0.01*	0.83	0.07	0.27
**medial orbitofrontal**	4.01	0.01	0.15	0.23	< 0.01*	0.98	0.48	0.22
**middle temporal**	12.48	< 0.01	0.15	< 0.01*	< 0.01*	0.10	< 0.01*	0.32
**parahippocampal**	4.05	0.01	0.92	0.66	0.01*	0.96	0.04*	0.07
**pars opercularis**	5.77	< 0.01	0.13	0.02*	< 0.01*	0.90	0.19	0.43
**pars triangularis**	6.43	< 0.01	0.37	< 0.01*	< 0.01*	030	0.07	0.74
**precuneus**	5.22	< 0.01	0.14	0.02*	< 0.01*	0.85	0.30	0.68
**rostral middle frontal**	4.84	< 0.01	0.83	0.24	< 0.01*	0.72	0.03*	0.16
**superior frontal**	3.05	0.03	0.47	0.36	0.01*	> 0.99	0.23	0.22
**superior temporal**	7.92	< 0.01	0.59	0.03*	< 0.01*	0.36	< 0.01*	0.13
**insula**	6.07	< 0.01	0.07	< 0.01*	< 0.01*	0.83	0.33	0.74

	one-way ANOVA		Tukey's multiple comparison					
	F value	p value	AD vs aMCI-m	AD vs aMCI-s	AD vs NC	aMCI-m vs aMCI-s	aMCI-m vs NC	aMCI-s vs NC
**caudal middle frontal**	4.36	< 0.01	0.24	0.55	< 0.01*	0.89	0.29	0.06
**entorhinal**	8.34	< 0.01	0.25	< 0.01*	< 0.01*	0.11	0.05	0.93
**fusiform**	12.15	< 0.01	0.05*	< 0.01*	< 0.01*	0.42	0.01*	0.13
**inferior parietal**	4.10	0.01	0.76	0.60	< 0.01*	> 0.99	0.07	0.07
**inferior temporal**	4.11	0.01	0.99	0.22	0.03*	0.32	0.03*	0.44
**isthmus cingulate**	3.20	0.03	0.35	0.73	0.02*	0.86	0.48	0.11
**lateral orbitofrontal**	2.98	0.04	0.89	0.59	0.03*	0.96	0.12	0.23
**lingual**	4.71	< 0.01	0.13	0.07	< 0.01*	> 0.99	0.34	0.33
**medial orbitofrontal**	4.23	< 0.01	0.31	0.66	< 0.01*	0.87	0.27	0.05*
**middle temporal**	3.98	0.01	0.86	0.32	< 0.01*	0.79	0.05	0.23
**parahippocampal**	3.70	0.02	0.95	0.91	0.02*	> 0.99	0.06	0.04*
**pars opercularis**	4.07	0.01	0.38	0.29	< 0.01*	> 0.99	0.19	0.16
**precuneus**	5.73	< 0.01	0.45	0.25	< 0.01*	> 0.99	0.04*	0.04*
**rostral middle frontal**	3.42	0.02	0.55	0.50	0.01*	> 0.99	0.22	0.15
**superior frontal**	3.45	0.02	0.61	0.43	0.01*	> 0.99	0.17	0.19
**superior temporal**	4.16	0.01	0.99	0.43	0.01*	0.54	0.02*	0.22
**supramarginal**	2.92	0.04	0.63	0.73	0.03*	> 0.99	0.28	0.14
**insula**	3.20	0.03	0.26	0.14	0.02*	> 0.99	0.53	0.42

*indicates significant after multiple comparison ($p < 0.05$).

Supplementary Table 2. Spearman correlation between cortical thickness and neuropsychological measurements for AD, aMCI-m, aMCI-s and NC groups.

cortical region	AVLT		MMSE		MoCA		TMT		BNT	
	r	p	R	P	r	p	r	p	r	p
entorhinal	0.54	< 0.01	0.40	< 0.01	0.45	< 0.01	-0.48	< 0.01	0.49	< 0.01
fusiform	0.55	0.01	0.51	< 0.01	0.54	< 0.01	-0.59	< 0.01	0.62	< 0.01
inferior parietal	0.44	< 0.01	0.34	< 0.01	0.39	< 0.01	-0.31	< 0.01	0.42	< 0.01
inferior temporal	0.53	< 0.01	0.45	< 0.01	0.46	< 0.01	-0.40	< 0.01	0.54	< 0.01
isthmus cingulate	0.29	< 0.01	0.42	< 0.01	0.36	< 0.01	-0.34	< 0.01	0.51	< 0.01
lateral orbitofrontal	0.46	< 0.01	0.35	< 0.01	0.46	< 0.01	-0.44	< 0.01	0.41	< 0.01
lingual	0.35	< 0.01	0.33	< 0.01	0.33	< 0.01	-0.35	< 0.01	0.37	< 0.01
medial orbitofrontal	0.42	< 0.01	0.41	< 0.01	0.41	< 0.01	-0.28	0.02	0.29	< 0.01
middle temporal	0.51	< 0.01	0.43	< 0.01	0.46	< 0.01	-0.50	< 0.01	0.59	< 0.01
parahippocampal	0.46	< 0.01	0.27	0.02	0.35	< 0.01	-0.27	0.02	0.32	< 0.01
pars opercularis	0.42	< 0.01	0.35	< 0.01	0.31	< 0.01	-0.28	0.02	0.37	< 0.01
pars triangularis	0.46	< 0.01	0.27	0.02	0.37	< 0.01	-0.35	< 0.01	0.41	< 0.01
precuneus	0.49	< 0.01	0.39	< 0.01	0.35	< 0.01	-0.34	< 0.01	0.47	< 0.01
superior parietal	0.44	< 0.01	0.38	< 0.01	0.39	< 0.01	-0.24	0.04	0.34	< 0.01
superior temporal	0.52	< 0.01	0.37	< 0.01	0.41	< 0.01	-0.41	< 0.01	0.56	< 0.01
supramarginal	0.43	< 0.01	0.27	0.02	0.29	0.01	-0.24	0.04	0.35	< 0.01
insula	0.59	< 0.01	0.33	< 0.01	0.41	< 0.01	-0.45	< 0.01	0.49	< 0.01

cortical region	AVLT		MMSE		MoCA		TMT		BNT	
	r	p	r	p	r	p	r	p	r	p
entorhinal	0.61	< 0.01	0.42	< 0.01	0.50	< 0.01	-0.53	< 0.01	0.56	< 0.01
fusiform	0.65	< 0.01	0.49	< 0.01	0.49	< 0.01	-0.49	< 0.01	0.62	< 0.01
inferior parietal	0.40	< 0.01	0.30	0.01	0.30	0.01	-0.23	0.05	0.36	< 0.01
inferior temporal	0.50	< 0.01	0.37	< 0.01	0.41	< 0.01	-0.40	< 0.01	0.48	< 0.01
lateral orbitofrontal	0.48	< 0.01	0.30	< 0.01	0.39	< 0.01	-0.33	< 0.01	0.33	< 0.01
lingual	0.33	< 0.01	0.25	0.03	0.26	0.03	-0.25	0.04	0.33	< 0.01
middle temporal	0.48	< 0.01	0.29	0.01	0.41	< 0.01	-0.35	< 0.01	0.45	< 0.01
parahippocampal	0.45	< 0.01	0.30	0.01	0.33	< 0.01	-0.25	0.04	0.32	< 0.01
pars opercularis	0.44	< 0.01	0.38	< 0.01	0.44	< 0.01	-0.24	0.04	0.30	< 0.01
precuneus	0.50	< 0.01	0.51	< 0.01	0.46	< 0.01	-0.35	< 0.01	0.48	< 0.01
superior temporal	0.52	< 0.01	0.29	0.01	0.37	< 0.01	-0.38	< 0.01	0.52	< 0.01

The table lists the high correlation cortical regions with corresponding neuropsychological measurements ($p < 0.05$). The above one is left hemisphere and the below one is right hemisphere.
